# Deletion at the 5’-end of Estonian ASFV strains associated with an attenuated phenotype

**DOI:** 10.1038/s41598-018-24740-1

**Published:** 2018-04-25

**Authors:** Laura Zani, Jan Hendrik Forth, Leonie Forth, Imbi Nurmoja, Simone Leidenberger, Julia Henke, Jolene Carlson, Christiane Breidenstein, Arvo Viltrop, Dirk Höper, Carola Sauter-Louis, Martin Beer, Sandra Blome

**Affiliations:** 1 Friedrich-Loeffler-Institut, Suedufer 10, 17493 Greifswald – Insel Riems, Germany; 2 Estonian Veterinary and Food Laboratory, Kreutzwaldi 30, 51006 Tartu, Estonia; 3 Institute of Veterinary Medicine and Animal Sciences, Estonian University of Life Sciences, Kreutzwaldi 62, 51014 Tartu, Estonia

## Abstract

African swine fever (ASF) was introduced into the Eastern European Union in 2014 and led to considerable mortality among wild boar. In contrast, unexpected high antibody prevalence was reported in hunted wild boar in north-eastern Estonia. One of the causative virus strains was recently characterized. While it still showed rather high virulence in the majority of experimentally infected animals, one animal survived and recovered completely. Here, we report on the follow-up characterization of the isolate obtained from the survivor in the acute phase of infection. As a first step, three *in vivo* experiments were performed with different types of pigs: twelve minipigs (trial A), five domestic pigs (trial B), and five wild boar (trial C) were inoculated. 75% of the minipigs and all domestic pigs recovered after an acute course of disease. However, all wild boar succumbed to infection within 17 days. Representative samples were sequenced using NGS-technologies, and whole-genomes were compared to ASFV “Georgia 2007/1”. The alignments indicated a deletion of 14560 base pairs at the 5’ end, and genome reorganization by duplication. The characteristic deletion was confirmed in all trial samples and local field samples. In conclusion, an ASFV variant was found in Estonia that showed reduced virulence.

## Introduction

In 2014, African swine fever virus (ASFV) was introduced into Poland and the Baltic European Union (EU) member states Latvia, Lithuania and Estonia. Since then, slow but constant spread of this notifiable disease has been observed^1^. With regard to outbreak characteristics, detection of fallen animals and virus prevails. However, in some regions, a different pattern in cause of the epidemic has been observed^1^. In the follow-up of those observations, we recently reported an animal experiment that aimed at the biological characterization of an ASFV strain from north-eastern Estonia, where an unexpectedly high ASFV-antibody prevalence was found in hunted healthy animals^2^. In this previous animal trial, ten wild boar were inoculated with the above mentioned ASFV strain to evaluate if the clinical course of the disease differed from infections with the so far known highly virulent Caucasian strains^3–6^. In brief, nine out of ten animals succumbed to the infection showing typical lesions. The surviving wild boar recovered completely and was slaughtered in good health status 96 days post infection (dpi). Comingling of the survivor with three sentinel wild boar from 50 dpi did not lead to disease transmission. Taken together, the virus showed still considerable virulence and lethality, but one animal recovered and could represent one of the antibody positive wild boar found in the hunting bags of north-eastern Estonia. These results left us with several unanswered questions, including: Is the survival of one animal within the normal range of clinical courses of a highly virulent ASFV strain or is it an indication for true attenuation? Could a further animal passage lead to a more attenuated phenotype? If there is attenuation, what is the genetic basis?

To address these questions and to further characterize the virus isolated from the surviving boar, three additional animal trials were performed to characterize the virus with different pig types. Since the survival rates and clinical courses were rather variable in the different trials, representative samples from each trial were full-genome sequenced using next-generation sequencing technologies and the resulting sequences were compared to ASFV “Georgia 2007/1” (FR682468.1). In order to confirm the circulation of the variant strain, Estonian field samples were screened for the mentioned mutation by *real-time* quantitative polymerase chain reaction (qPCR).

## Results

### Clinical course and pathomorphological findings

In all trials, the animals showed unspecific clinical signs during the first 10 days after oronasal inoculation.

In trial A (12 minipigs), all animals developed transient high fever (up to 41 °C on day 7 pi). The minipigs also showed transient anorexia and lethargy. One minipig (#69) was found dead the day after blood sampling (8 dpi). Necropsy revealed a mild pericarditis and atelectasis in the left lung. Two animals (#72 and #67) had to be euthanized due to severe respiratory distress (8 dpi and 15 dpi). Animal #72 showed lung edema and several hemorrhagic lymph nodes in necropsy. The other nine minipigs recovered completely and were slaughtered in good health at 36 dpi. The post-mortem examination revealed that two of the recovered minipig sows (#61 and #70) were pregnant around the 45^th^ day of gestation according to the size of the fetuses. The fetuses did not show any pathological findings indicative for ASF or any other disease while one of the pregnant sows (#61) presented a pericarditis. None of the other recovered pigs did have any visible lesions.

**Figure 1:**
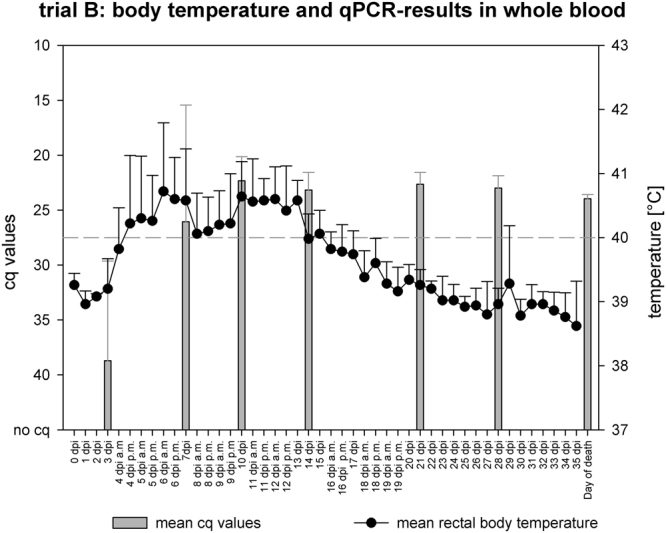
Trial B; body temperature and qPCR results in whole blood; grey bars indicate the mean cq-values at the sampling days; mean rectal body temperature is graphed as line and scatter plot; medium-dashed line represents the fever-cutoff (40 °C); in case of “dpi a.m./p.m.” rectal temperature was assessed twice a day, upper standard deviation is shown in error bars.

In trial B (5 domestic pigs), four out of five pigs started showing mild clinical signs such as lethargy, reduced feed intake and increased body temperature 4 to 6 dpi. Animal #98 reacted slightly later on day 10 pi and started with unspecific clinical signs like the other pigs. The second week after inoculation, the animals showed more severe clinical signs with transient high fever (see Fig. 1), reduced liveliness and responsiveness, and transient anorexia. At 17 dpi, animal #97 showed short-term (less than 24 hours) cyanosis on the acra (ears, mouth and tail). After this acute phase, all pigs recovered. From 19 dpi on, no clinical signs were observed, apart from animal #98 with a single body temperature peak at 29 dpi. Apart from one pig (#99) with fibrinous pericarditis, the post-mortem examination did not reveal any pathological findings indicative for an ASFV infection.

In trial C (5 wild boar), the animals developed unspecific clinical signs such as reduced feed intake and lethargy at 3–4 dpi. One female adult wild boar (#1) was found dead the morning after blood sampling (8 dpi). The next day, the second adult female (#3) was found dead and the male adult wild boar (#8) was euthanized due to severe respiratory distress. None of the animals had been near the humane endpoint the previous evening preceding death or euthanasia. The piglets survived until day 16 dpi (#81) and 17 dpi (#82) when they were euthanized reaching the humane endpoint. Necropsy revealed typical findings for an acute ASFV infection such as lung edema, hemorrhagic lymph nodes and petechiae in the renal cortex.

### Detection of virus and viral genome

**Figure 2:**
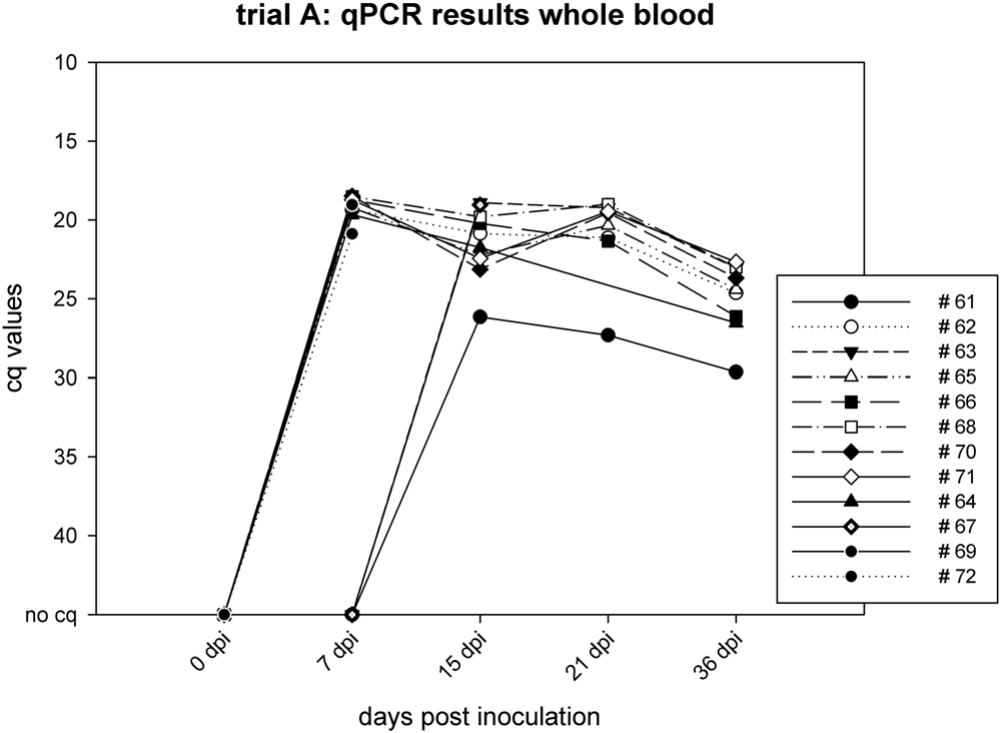
Trial A; qPCR results whole blood cq values graphed as line and scatter plot.

In trial A, the first animals started yielding positive qPCR results in whole blood samples from 7 dpi on. All animals were positive for ASFV genome in qPCR at day 15 pi and the recovering minipigs showed stable genome loads until the end of the trial (see Fig. 2). In organ pools of the fetuses of two pregnant sows (#61 and #70), no ASFV genome was detectable by qPCR while the sows showed positive qPCR results in whole blood on the day of necropsy. Regarding the tissue samples, minipigs that died during the acute phase of the disease yielded much higher viral genome loads in most organs compared to the recovered minipigs (see supplementary Table 1). The oropharyngeal and fecal swabs showed single weakly (quantitation cycle (cq) value > 35) or moderately (cq value > 22) positive qPCR results during the acute phase of the disease.

Results of the haemadsorption test of the sera were corresponding to the samples with detectable virus genome at day 7 and 15 pi. At the day of necropsy, serum samples of the animals that died during the acute phase reacted positive in the haemadsorption test, while the sera of the recovered pigs were negative. The tissue samples of the recovered minipigs showed positive results in lungs or tonsils while all other organs were negative for virus isolation (see supplementary Table 2).

In trial B, on 3 dpi two animals started with positive results in whole blood tested in qPCR. On 7 dpi, all pigs but animal #98 were tested positive in whole blood by qPCR and from 10 dpi on, ASFV genome was detectable in the whole blood of all pigs until the end of the trial (see Fig. 1). At the day of necropsy (36 dpi), samples of spleen and tonsils of all pigs except animal #100 showed weak positive results in qPCR. The tissue samples of the different lymph nodes and the salivary gland showed sporadic weak positive qPCR results in different pigs, whereas no ASFV genome was detectable in lung tissues (see supplementary Table 1). The oropharyngeal and fecal swabs showed single weakly (cq values > 35) positive qPCR results during the acute phase of the disease (see supplementary Fig. 1a + b).

The haemadsorption test of the serum from 3 dpi reflects these results with one clearly positive result from animal #99 and a doubtful result of animal #97. On 7 dpi, all pigs with positive qPCR results in whole blood showed clearly positive haemadsorption phenomena in serum samples. The serum of animals #97 and #100 reacted positive in the haemadsorption test while the serum of animal #60 was negative. From animals #98 and #99 no serum samples were taken due to their critical health status during the acute phase of the disease. Two weeks after the inoculation, serum of all pigs reacted positive in the haemadsorption test. Subsequently, at 21 dpi the serum of two pigs (#99, #100) showed negative results in the haemadsorption test. At 28 dpi, again animal #100 was the only animal reacting positive for hemadsorption using serum, and only three tissue samples were positive for virus isolation (see supplementary Table 2).

In trial C, on 7 dpi all wild boar were highly positive for viral genome in qPCR reaching a mean cq value of 17 in whole blood. The tissue samples of the three adult wild boar were all tested positive in qPCR. The tissue samples of the two piglets showed overall lower genome loads and some organs of animal #81 were tested negative for viral genome in the qPCR assays (see supplementary Table 1). Homogenized spleen samples showed high titers (10^4.5^–10^5.5^ haemadsorbing units (HAU)/mL) for the three adult wild boar. The two piglets yielded lower titers around 10^2^–10^3^ HAU/mL in their spleen suspensions. The same distribution was seen in the haemadsorption assay of lung and tonsil tissue: titers from 10^3.25^–10^4.25^ HAU/mL in the three adult wild boar while the same tissues were negative for haemadsorption in the two piglets (see supplementary Table 2). The blood samples from the day of necropsy showed high titers from 10^5.25^ HAU/mL (#1) to 10^7.25^ HAU/mL (#8).

### Detection of antibodies against ASFV

**Figure 3:**
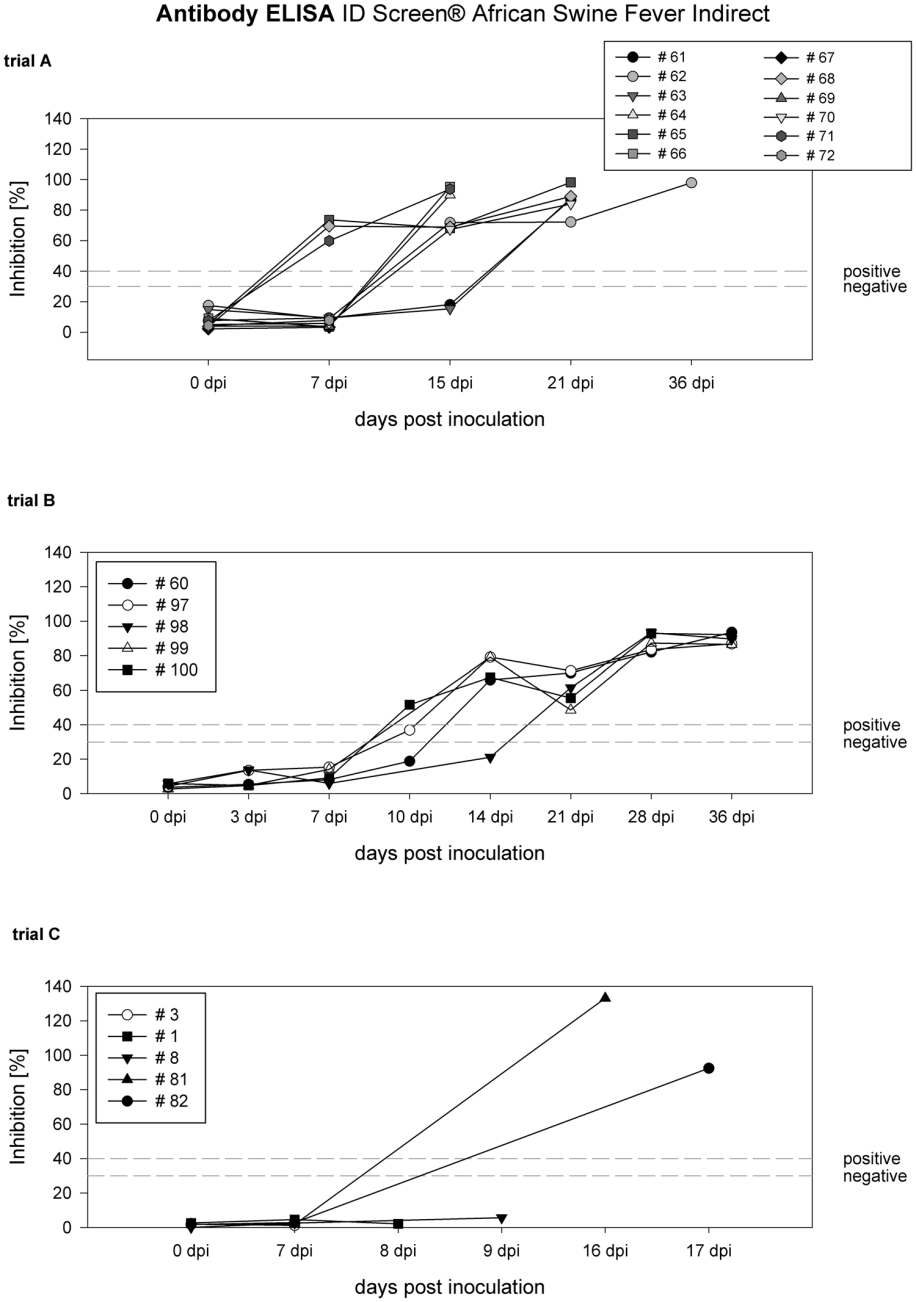
Antibody response trial A-C ELISA results in [%] inhibition graphed as line and scatter plots.

In trial A, 15 dpi four minipigs were tested positive for antibodies against ASFV and at 21 dpi all but one of the recovered minipigs showed positive enzyme-linked immunosorbent assay (ELISA) results. At the necropsy on the end of the trial, all nine minipigs that survived the acute phase of the disease were still positive for ASFV-specific antibodies (see Fig. 3).

The pigs of trial B showed the first positive ELISA results on 10 dpi and two weeks after the inoculation four out of five pigs were tested clearly positive for antibodies against ASFV. From 21 dpi until the end of the trial at 36 dpi, all animals were tested positive for antibodies (Fig. 3).

The wild boar in trial C showed negative ELISA results in samples from 7 dpi. The sera taken from the two piglets at the necropsy showed positive results (see Fig. 3), while the sera from the adult wild boar were still negative for antibodies at their endpoints.

### Next-generation sequencing (NGS)

**Figure 4:**
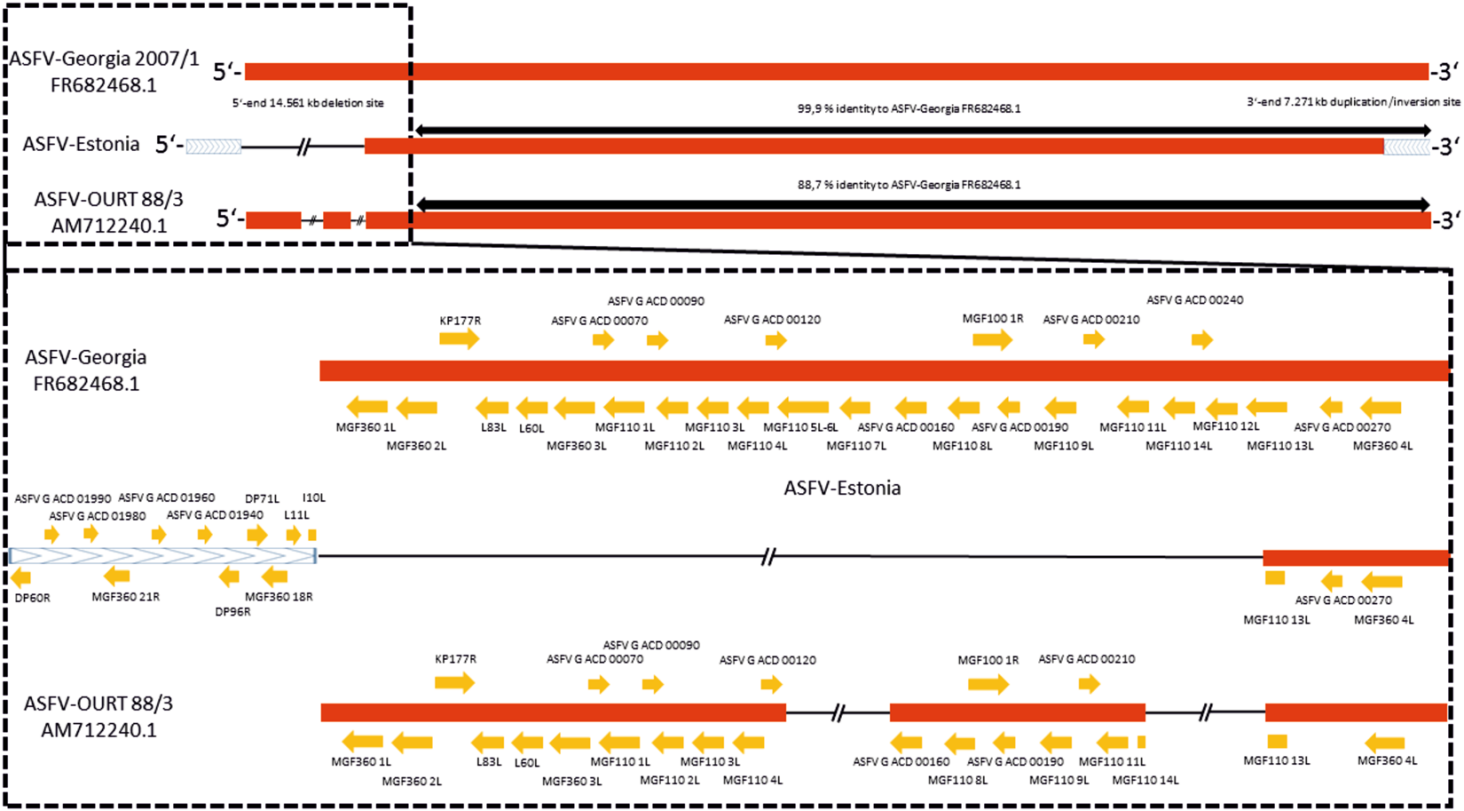
Deletion and reorganization site. Overview of the ASFV Estonia reorganization sites and comparison with the respective region (15 kb from the 5′ end) of ASFV-Georgia 2007/1 and the natural attenuated ASFV OURT 88/3 (only considering shared deletions).

For four out of five samples (inocula for animal trials and trial samples), the full ASFV-genome sequence could be assembled. The genomes comprise 182,446 base pairs (bp) with an overall sequence identity of 99.99% among themselves. In comparison to the reference genome ASFV “Georgia 2007/1” (FR682468.1), which comprises 189,344 bp, the first 14,560 bp at the 5′ end are missing. This deletion results in the loss of 26 complete genes including I83L, I60L and KP177R as well as members of the MGF110 (1L-14L), MGF360 (1L-3L), and the partial MGF110 13 L gene. Furthermore, 7271 bp from the 3′ end were found to be inversely bound at the 5′ end leading to the duplication of 10 complete genes including members of the MGF360 (18 R and 21 R) and L11L as well as one partial gene I10L (Fig. 4). In comparison to FR682468.1, the sequence identity of the core genome of ~175 kB amounts to 99.9%, thereby not considering the very different 5′ end and a 344 bp longer tail at the 3′-end.

### Confirmation of deletion site and field sample screening

Representative samples from all animal experiments including the initial wild boar trial^2^ and the original Estonian field sample were examined by tailored PCR and Sanger sequencing. The deletion site was confirmed in all samples (see supplementary Fig. 2).

**Figure 5:**
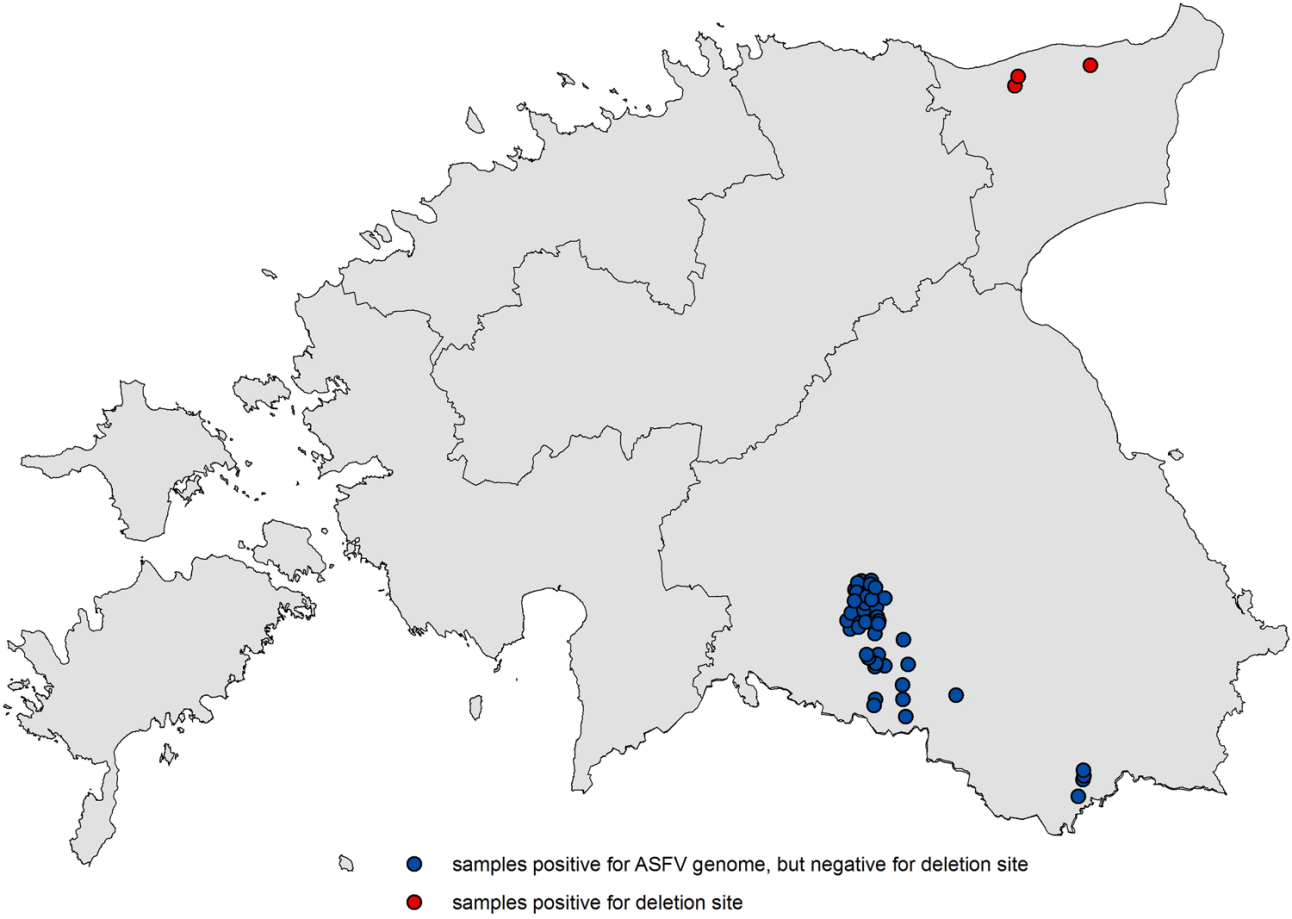
Map of Estonia including the results of the field sample screening. The screening included 61 original field samples from 2014 provided by Estonian Veterinary and Food Laboratory.

Sixty-one Estonian field samples from 2014 were screened for the characteristic deletion site and three samples were tested positive for the mutation by qPCR. All three samples were from Ida-Viru county in north-eastern Estonia and one of them was the original field sample used in the first trial (see map in Fig. 5). The other 58 samples were tested positive for the ASFV wild-type sequence.

## Discussion

**Figure 6:**
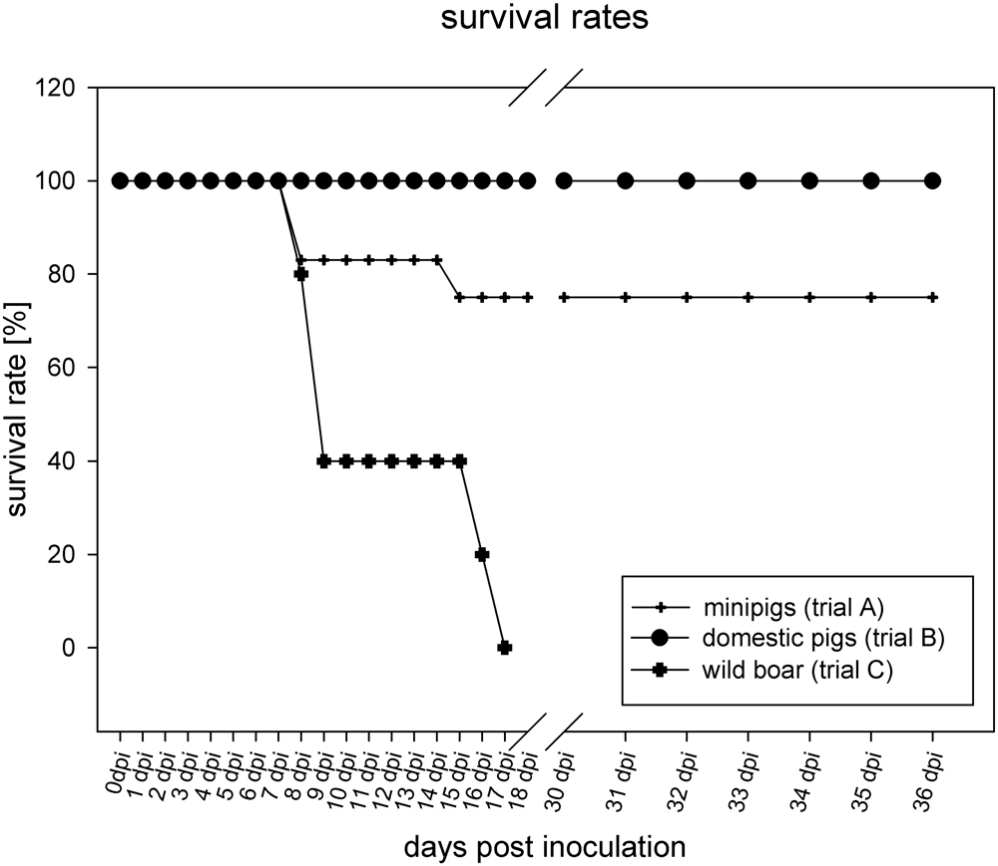
Survival rates trial A–C survival rates of the inoculated animals in [%] graphed as line and scatter plot.

**Figure 7:**
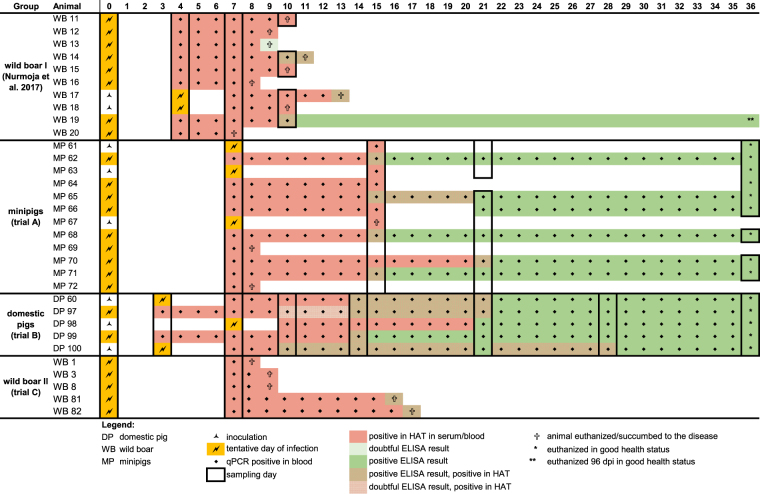
Overview of antibody response, disease course and viremia data between sampling days was assumed.

Compared to what is so far known about the virulence of ASFV genotype II in both domestic pigs and European wild boar under experimental conditions^3–7^, the north-eastern Estonian strain re-isolated from a surviving animal during acute infection showed a clearly attenuated phenotype in trials A and B. After all pigs developed acute clinical disease, these trials ended with survival rates between 75% and 100% (see Figs 6 and 7).

The deaths of three minipigs in trial A were not clearly linked to ASFV infection and could as well be consequence of their high stress sensitivity and the invasive sampling procedures during the acute phase of the disease. The use of minipigs with potbelly pig ancestry for ASF trials with blood sampling has to be reassessed for animal welfare reasons. In our experience, stress responses were much less pronounced in domestic pigs and even in (tame) wild boar. In both trials, all recovering animals showed seroconversion that was detectable by all routine diagnostic methods. The first antibody responses were detected at day 10 pi (trial B) and 15 dpi (trial A), respectively. This matches the results of previous studies^2,7^. However, there are not many reports about the time point of seroconversion of ASFV genotype II infected animals because up to now in most trials the animals died before the development of an antibody response. The fecal and oral shedding of ASFV genome was quite low compared to the genome load in blood samples and limited to the acute phase of the disease. This agrees with results of previous experiments^3,7^. With regard to gastrointestinal signs in general, only slight obstipation was observed in the febrile phase of infection, and macroscopically no blood admixture was seen. Based on the observed detection frequency and the low viral genome load, the suitability of fecal and oral swab samples for reliable and timely detection of the disease has to be questioned. The qPCR-negative results of the minipig fetuses indicate that transplacental transmission did not occur over the whole study period. Given the estimated stage of gestation (roughly 45 days), the mothers were inoculated in very early pregnancy and did not transmit the virus over 36 days of infection. This is in line with unpublished field observations but not with a case report from Nigeria^8^. However, under our experimental conditions, all fetuses remained negative and did not show any negative effects related to the infection of the mothers.

The outcome of trial C is in contrast to the other trials. However, it reflects more or less the disease course in the initial wild boar trial^2^ in which all but one wild boar succumbed to the infection. This could lead to the assumption that wild boar are more susceptible to infection with this ASFV strain than domestic pig breeds which is not in accordance with the literature^5^ and is also only partially in line with the field observations that showed several apparently healthy, but sero-positive wild boar in the hunting bag of north-eastern Estonia. The slightly higher inoculation dose in trial C is not a sufficient explanation for the higher mortality either, since previous studies^5^ did not reveal a measurable dose dependency. However, the piglets survived at least until 16 and 17 dpi. Therefore, it could be discussed if they are more resistant compared to the adult wild boar, which is inconsistent with former studies on ASFV Armenia^4^ but fits with the observation that the detection of antibodies was more likely in the young age class^1^. In general, a negative influence of the necessary immobilization of the adult animals during the acute phase of the disease has to be taken in consideration. However, this had no influence in previous trials but harmonized experiments targeting the direct comparison of the clinical course together with the assessment of immunological parameters in domestic pigs and wild boar, infected with the variant strain, would be required to finally clarify this issue.

The attenuated disease course shown especially in trials A and B can be associated with the results of the full-genome sequencing.

Among the 26 genes missing from the viral genome, thirteen belong to the multigene family MGF110 (1L-14L)^9,10^ and three to the MGF360 (1L-3L). While the specific functions of these genes are unknown^11^, it was shown that members of the MGF110 carry C-Terminal KDEL endoplasmic reticulum retention motifs and might be involved in preparing the ER for viral morphogenesis^12^. Although the respective MGF360 1L-3L genes are not characterized and their function is also unknown, other MGF360 members were found to be important for ASFV replication in ticks^13^, macrophages^14,15^ as well as domestic pigs^16–19^.

Further deleted genes include MGF100 1R, L83L, L60L and KP177R. While for the first three, no function is known^11^, the KP177R genes encodes for the early membrane protein P22^20^. The mechanism by which this major genome re-organisation occurred remains unclear. Nonetheless, the 5′ end deletion as well as the duplication and inverse binding of ~7 kb from the 3′ to the 5′ end could be explained by a false separation of head-to-tail concatamers during viral DNA replication^21^.

Whether the duplication of ten genes including DP71L and DP96R, two genes which were previously reported as important for virulence^11,22,23^ and the uncharacterised genes MGF360 18R, MGF360 21R, L11L and DP60R as well as the partial duplication of the I10L gene, which codes for a P22 homologue, has an effect on virulence remains to be investigated.

A direct comparison of the ASFV Estonia strain with the naturally attenuated ASFV strain OURT 88/3 reveals some similarities (see Fig. 4) but also major differences. While both strains lack members of the MGF110 (4L–7L and 12–13L)^24,25^, the major deletions are at different positions. While the main changes of the Estonian strain are at the true 5′-end of the coding sequence, the major deletion of OURT88/3 is further downstream concerning e.g. members of the MGFs 306 and 505. Yet, one can speculate that the shared deletions are already part of the attenuation process.

It could be hypothesized that large-scale mutations could occur more often but an ASFV strain that has an attenuated phenotype with lower mortality rates in swine, in the absence of a reservoir vector, will probably vanish due to the animals clearing the virus before it is transmitted via bloody excretions or the dead animal’s carcass. The low case number and the limited geographical distribution of the sub-genotype in 2014 can substantiate this hypothesis: all three samples positive for the deletion site are located in Ida-Viru county, around 200 kilometers from the outbreak in southern Estonia (see map in Fig. 5). At the same time, two of the wild boar infected with the sub-genotype were tested positive for ASFV-specific antibodies, a fact that fully supports the theory of an attenuated phenotype.

The question whether the variant strain occurred in the local wild boar population by spontaneous mutation or was introduced from somewhere else, remains unanswered and needs further investigation.

However, the presence of attenuated phenotypes, leads to new challenges regarding surveillance of wild boar population as well as domestic pig farms. The observed inconspicuous clinical signs and low mortality of the animals in the first two trials could easily go unnoticed under common pig farm conditions. Thus, farmers and veterinarians should be sensitized to suspect ASF not only if severe signs are observed. The occurrence of almost silent infections with mild and unspecific signs carries the risk of undetected disease spread and surveillance should be adapted accordingly. In this context, it might be reasonable to sample not only wild boar carcasses in ASF-free areas, but also to test hunted wild boar. Otherwise the occurrence of such an attenuated ASFV-subtype could be missed.

## Materials and Methods

### Experimental design

The study comprised three animal experiments (trials A, B and C) that were carried out to collect suitable reference materials and to assess virulence and pathogenesis characteristics of genotype II ASFV from Estonia upon animal passaging. To this end, an ASFV-positive blood suspension was prepared that contained ethylenediaminetetraacetic acid (EDTA)-treated blood samples from a wild boar with acute-transient ASF^2^ diluted in phosphate-buffered saline (PBS) to a final titer of app. 10^5^ HAU per mL. The suspension was used to inoculate the pigs in trial A and B.

Trial A comprised 12 sub-adult minipigs of both sexes from the breeding unit at the Friedrich-Loeffler-Institut (FLI) aged approximately six months at the start of the trial. For the experiment, the animals were moved from the FLI quarantine stables into the high containment facilities (L3+) where they were kept together in one pen. All animals were individually ear-tagged with numbers #61 to #72. Over the course of the trial, the animals were fed a commercial pig food with hay cob supplement and had access to water *ad libitum*. After an acclimatization phase, the minipigs were oronasally inoculated with 2 mL of the above-mentioned blood suspension using a single-use syringe without needle. Clinical parameters of all animals were assessed daily based on a harmonized scoring system as previously described^5^. In brief, parameters like temperature (assessed only on sampling days), anorexia, recumbency, skin alterations (cyanosis, haemorrhages, necrosis), joint lesions, breathing, ocular discharge, digestion, and neurological disorders were assigned points according to the severity of findings. The sum of the points was recorded as the clinical score (CS) that was also used to define humane endpoints. Over the course of the trial, levels of viremia, virus distribution, virus shedding, and antibody responses were assessed. For this purpose, blood samples were collected along with oropharyngeal and fecal swabs at days 0, 7, 15, 21 dpi and at the end of the trial (36 dpi). Animals reaching the humane endpoint or that were suffering unacceptably without reaching the endpoint were euthanized through intracardial injection of embutramide (T61, Merck) after deep sedation with tiletamine/zolazepam (Zoletil®, Virbac). Necropsy was performed on all animals, and at the same time, tissue samples (lymph nodes, spleen, tonsil, salivary gland and lung), blood (EDTA, serum) and swab samples were collected for reference purposes.

Trial B comprised five commercial domestic pigs aged approximately six months at the start of the trial. All animals were individually ear-tagged upon arrival with numbers #60 and #97 to #100. After an acclimatization phase under the husbandry conditions detailed above, the animals were oronasally inoculated with 2 mL of the above-mentioned blood suspension. As in trial A, clinical monitoring took place on a daily basis, and levels of viremia, virus distribution, virus shedding, and antibody responses were assessed.

For this purpose, blood samples were collected along with oropharyngeal and fecal swabs at 0, 3, 7, 10, 14, 21, 28 dpi and at the end of the trial at 36 dpi. All animals were slaughtered 36 dpi (exsanguination after electro-stunning) and necropsy was performed. Again, tissue samples (lymph nodes, spleen, tonsil, salivary gland and lung), blood (EDTA, serum) and swab samples were collected for reference purposes.

Trial C comprised five wild boar from the breeding unit at the FLI of different sexes and ages (three adult wild boar around two years old and two piglets app. 6 months old). The wild boar were immobilized with an intramuscular injection of tiletamine/zolazepam (Zoletil®, Virbac) and moved to the high-containment facilities. They were individually eartagged (#1, #3, #8, #81, and #82) and oronasally infected with 2 mL blood suspension (titer of app. 10^6.5^ HAU/ mL) from trial B (a mixture of blood samples from different pigs).

As in trials A and B, clinical monitoring took place on a daily basis, apart from the rectal body temperature assessment, due to working safety conditions. Levels of viremia, virus shedding, and antibody responses were assessed. For this purpose, blood samples were collected along with oropharyngeal and fecal swabs at day 0 and day 7 pi. Since it was necessary to immobilize the wild boar to take blood samples, the sampling time points were reduced to a minimum. Animals reaching the humane endpoint or that were suffering unacceptably without reaching the endpoint were euthanized through exsanguination after deep sedation with tiletamine/zolazepam (Zoletil®, Virbac). Necropsy was performed on all animals and tissue samples (lymph nodes, spleen, tonsil, salivary gland and lung) and blood (EDTA, serum) were collected for reference purposes.

In all trial parts, all applicable animal welfare regulations, including EU Directive 2010/63/EC and institutional guidelines, were taken into consideration. The animal experiments were approved by the competent authority (Landesamt für Landwirtschaft, Lebensmittelsicherheit und Fischerei (LALLF) Mecklenburg-Vorpommern) under reference number LALLF 7221.3-2-023/15.

### Cells

Blood for the preparation of Peripheral Blood Mononuclear Cells (PBMC)-derived macrophages was collected from healthy domestic donor pigs that are routinely kept at the FLI. In brief, PBMCs were obtained from EDTA-treated blood using Pancoll Animal density gradient medium (PAN Biotech). PBMCs were grown in RPMI-1640 cell culture medium with 4-(2-hydroxyethyl)−1-piperazineethanesulfonic acid (HEPES) and 10% fetal calf serum (FCS) at 37 °C in a humidified atmosphere containing 5% CO_2_. The medium was supplied with amphotericin B, streptomycin and penicillin to avoid bacterial and fungal growth. To facilitate maturation of macrophages, granulocyte macrophage colony-stimulating factor (GM-CSF) was added to the cell culture medium at 2 ng/mL.

### Laboratory investigations

#### Processing of samples

Oropharyngeal swabs were soaked in 1 mL of medium (EMEM without addition of FCS), vortexed for app. 15 seconds, incubated for one hour at room temperature, and decanted in microcentrifuge tubes. Serum samples, which were obtained from native blood by centrifugation at 2500 × g for 20 minutes at 20 °C, were aliquoted and stored at −80 °C until further use. Tissue samples of tonsil, spleen, salivary gland, lung, and lymph nodes were collected at necropsy and stored at −80 °C. For qPCR and virus isolation (haemadsorption tests), tissue samples were homogenized with a metal bead in 1 mL phosphate-buffered saline (PBS) using a TissueLyser II (Qiagen).

#### Virus detection

For qPCR, viral nucleic acid was extracted using the QIAamp® RNA Viral Mini Kit (Qiagen) or the NucleoMagVet-Kit (Macherey-Nagel) and the KingFisher® extraction platform (Thermo Scientific). Both extraction methods were slightly modified through the addition of an internal control DNA. The nucleic acid extraction was performed with 75 µl of whole blood and 150 µl of organ homogenate and swab material. Subsequently, qPCR was performed according to the protocol published by King *et al*.^26^ with slight modifications. For confirmation, the virotype ASFV PCR Kit (Qiagen) was employed according to the manufacturer’s instructions. Results of both qPCRs were recorded as quantification cycle (cq) values.

To detect ASFV in serum and tissue samples, a haemadsorption test (HAT) was carried out using PBMC-derived macrophages according to slightly modified standard procedures^27^. In brief, isolated PBMCs were seeded into a 96-well microplate at a density of 1.9 × 10^6^ cells/mL, 100 μL per well. After 16–24 hours, non-adherent cells were removed and cell culture medium containing GM-CSF was replenished. The culture was then incubated for 24 to 48 hours to allow initial maturation of macrophages. Subsequently, 20 µl of serum samples and 30 µl of organ homogenate were added to each well. Tests were performed in duplicates. When using organ homogenates, cells were washed after 2 hours adsorption time using lukewarm PBS, whereas serum was left on the cells until the evaluation of the test. After 24 hours of incubation 20 µl of homologues 1% erythrocyte suspension was added to each well. For readout, cultures were analyzed for haemadsorption phenomena over a period of two days. Doubtful results were confirmed by an additional passage. Virus titration was performed by endpoint titration of the diluted blood suspensions. In this case, the PBMC preparation was seeded into 96-well microplates, the test volume was 100 µl per dilution step and 20 µl of a 1% homologous erythrocyte suspension was added. These samples were tested in quadruplicate.

#### Antibody detection

For the detection of antibodies against African swine fever virus, two commercial ELISA kits were carried out following the manufacturer’s instructions (Ingezim PPA COMPAC, Ingenasa; ID SCREEN African swine fever virus INDIRECT, IDvet). The Ingezim PPA ELISA detects antibodies directed against p72 in a competitive format. The ID SCREEN is an indirect ELISA using antigens p32, p62 and p72. All serum samples were tested in duplicate.

### Full-genome sequencing

#### Sample preparation

The material for the full-genome sequencing was gained by salting-out of viral DNA^28^ after propagation of the virus on PBMC-derived macrophages to avoid high loads of swine related DNA. Therefore, the protocol was slightly modified regarding a shortened incubation time (60 minutes at 37 °C) and the addition of RNase A (10 mg/ml) in the first step instead of proteinase K (10 mg/mL), which was added in the next step and incubated for 60 minutes at 56 °C.

#### Sequencing

For full genome sequences, 500 ng of input material were fragmented using Covaris M220 Focused-ultrasonicator™ (Covaris), and ligated to suitable Illumina adapters (NEXTflex-96™ DNA Barcodes, BiooScientific) using a SPRI-TE library system (Beckman Coulter) with SPRIworks Fragment Library Cartridges II (for Roche FLX DNA sequencer; Beckman Coulter). Size exclusion was performed manually with AMPure XP magnetic beads in two steps for a final size distribution of 500–600 bp long fragments. After quality control of the libraries on a Bioanalyzer 2100 (Agilent Technologies), the libraries were quantified using using Kapa Library Quantification Kit for Illumina platforms (Kapa Biosystems), pooled and sequenced on a MiSeq instrument (Illumina) with MiSeq reagent Kit v3 in 2 × 300 bp PE mode (Illumina). For data analysis, the reads were mapped against the nearest reference genome (Newbler v3.0, Roche). All mapped reads were extracted and de novo assembled (Newbler v3.0, Roche). Since this approach delivered three or more contigs, the software ContigGraph (unpublished) was used to determine the connections of single contigs for manual assembly of the full genome. Afterwards the whole data set was mapped against the full genome (Newbler v3.0, Roche).

#### Sanger sequencing, PCR and qPCR screening

All nucleic acids for PCR and qPCR were extracted using the High Pure DNA Template Preparation Kit (Roche) or the QIAamp® RNA Viral Mini Kit (Qiagen) according to the manufacturer’s instructions. For classical PCR, Phusion Green Hot Start II High-Fidelity PCR Mastermix (Thermo-Scientific) and for qPCR, QuantiTect Multiplex PCR NoROX Kit (Qiagen), were used according to the manufacturer’s instructions.

For classical PCR and sanger sequencing, primers were designed to amplify either the reorganised or the wild type sequence by placing them overlapping the reorganisation site.

For qPCR screening, tailored primers were designed amplifying short DNA-fragments of the reorganisation site with an additional Taqman-probe inside the target fragments. The tested field samples were provided by the Estonian Veterinary and Food Laboratory. The panel of 61 samples contained blood, spleen and bone marrow specimens from Estonian wild boar collected during the outbreak situation in 2014.

All primers and probes were designed using Geneious v. 10.0.9 (Supplementary Table).

All data were recorded and evaluated using Microsoft Excel 2010 (Microsoft Deutschland GmbH) and SigmaPlot for Windows version 11.0 (Systat Software, Inc.)

### Data availability

All sequence data was uploaded to the European Nucleotide Archive (EMBL-EBI) under the study accession number PRJEB24381.

## Electronic supplementary material

Supplementary Dataset 1

## References

[1] Nurmoja I (2017). Development of African swine fever epidemic among wild boar in Estonia - two different areas in the epidemiological focus. Scientific reports.

[2] Nurmoja, I. *et al*. Biological characterization of African swine fever virus genotype II strains from north-eastern Estonia in European wild boar. *Transboundary and emerging diseases*, 10.1111/tbed.12614 (2017). 10.1111/tbed.12614 28116841

[3] Gabriel C (2011). Characterization of african Swine Fever virus caucasus isolate in European wild boars. Emerging infectious diseases.

[4] Blome S, Gabriel C, Dietze K, Breithaupt A, Beer M (2012). High virulence of African swine fever virus caucasus isolate in European wild boars of all ages. Emerg Infect Dis.

[5] Pietschmann J (2015). Course and transmission characteristics of oral low-dose infection of domestic pigs and European wild boar with a Caucasian African swine fever virus isolate. Archives of virology.

[6] Gallardo C (2015). Experimental Infection of Domestic Pigs with African Swine Fever Virus Lithuania 2014 Genotype II Field Isolate. Transbound Emerg Dis.

[7] Guinat C (2014). Dynamics of African swine fever virus shedding and excretion in domestic pigs infected by intramuscular inoculation and contact transmission. Vet Res.

[8] Antiabong J (2006). Molecular Evidence Of Transplacental (Vertical) Route Of Transmission Of African Swine Fever In Foetus Of Pig: A Case Report. The Internet Journal of Veterinary Medicine.

[9] Pires S, Ribeiro G, Costa JV (1997). Sequence and organization of the left multigene family 110 region of the Vero-adapted L60V strain of African swine fever virus. Virus genes.

[10] Almendral JM, Almazan F, Blasco R, Vinuela E (1990). Multigene families in African swine fever virus: family 110. Journal of virology.

[11] Dixon LK, Chapman DA, Netherton CL, Upton C (2013). African swine fever virus replication and genomics. Virus research.

[12] Netherton C, Rouiller I, Wileman T (2004). The Subcellular Distribution of Multigene Family 110 Proteins of African Swine Fever Virus Is Determined by Differences in C-Terminal KDEL Endoplasmic Reticulum Retention Motifs. Journal of virology.

[13] Burrage TG, Lu Z, Neilan JG, Rock DL, Zsak L (2004). African swine fever virus multigene family 360 genes affect virus replication and generalization of infection in Ornithodoros porcinus ticks. Journal of virology.

[14] Zsak L (2001). African swine fever virus multigene family 360 and 530 genes are novel macrophage host range determinants. Journal of virology.

[15] Neilan JG (2002). Novel Swine Virulence Determinant in the Left Variable Region of the African Swine Fever Virus Genome. Journal of virology.

[16] Reis AL (2016). Deletion of African swine fever virus interferon inhibitors from the genome of a virulent isolate reduces virulence in domestic pigs and induces a protective response. Vaccine.

[17] Golding JP (2016). Sensitivity of African swine fever virus to type I interferon is linked to genes within multigene families 360 and 505. Virology.

[18] O’Donnell V (2015). African Swine Fever Virus Georgia Isolate Harboring Deletions of MGF360 and MGF505 Genes Is Attenuated in Swine and Confers Protection against Challenge with Virulent Parental Virus. Journal of virology.

[19] O’Donnell V (2016). African swine fever virus Georgia isolate harboring deletions of 9GL and MGF360/505 genes is highly attenuated in swine but does not confer protection against parental virus challenge. Virus research.

[20] Camacho A, Vinuela E (1991). Protein P22 of African Swine Fever Virus - an Early Structural Protein That Is Incorporated into the Membrane of Infected-Cells. Virology.

[21] Rojo G, Garcia-Beato R, Vinuela E, Salas ML, Salas J (1999). Replication of African swine fever virus DNA in infected cells. Virology.

[22] Rivera J (2007). The MyD116 African swine fever virus homologue interacts with the catalytic subunit of protein phosphatase 1 and activates its phosphatase activity. Journal of virology.

[23] Zhang F, Moon A, Childs K, Goodbourn S, Dixon LK (2010). The African swine fever virus DP71L protein recruits the protein phosphatase 1 catalytic subunit to dephosphorylate eIF2alpha and inhibits CHOP induction but is dispensable for these activities during virus infection. Journal of virology.

[24] Chapman DA, Tcherepanov V, Upton C, Dixon LK (2008). Comparison of the genome sequences of non-pathogenic and pathogenic African swine fever virus isolates. The Journal of general virology.

[25] Sanchez-Cordon PJ (2016). Different routes and doses influence protection in pigs immunised with the naturally attenuated African swine fever virus isolate OURT88/3. Antiviral research.

[26] King DP (2003). Development of a TaqMan PCR assay with internal amplification control for the detection of African swine fever virus. J Virol Methods.

[27] Carrascosa, A. L., Bustos, M. J. & de Leon, P. Methods for growing and titrating African swine fever virus: field and laboratory samples. *Current protocols in cell biology/editorial board, Juan S. Bonifacino…* [*et al*.] Chapter 26, Unit26 14, 10.1002/0471143030.cb2614s53 (2011). 10.1002/0471143030.cb2614s53 22161547

[28] Miller SA, Dykes DD, Polesky HF (1988). A simple salting out procedure for extracting DNA from human nucleated cells. Nucleic acids research.

