# Supplementary material for: Deletion at the 5’-end of Estonian ASFV strains associated with an attenuated phenotype
Source: Sci Rep. 2018 Apr 25;8:6510. doi: 10.1038/s41598-018-24740-1 (PMC5916933; doi:10.1038/s41598-018-24740-1)
Supplement: Supplementary file 1 — Supplementary Dataset 1 [file 41598_2018_24740_MOESM1_ESM.docx]

Supplementary figures and tables

**Deletion at the 5’-end of Estonian ASFV strains associated with an attenuated phenotype**

Laura Zani^1^, Jan Hendrik Forth^1,^ Leonie Forth^1^, Imbi Nurmoja^2,3^, Simone Leidenberger^1^, Julia Henke^1^, Jolene Carlson^1^, Christiane Breidenstein^1^, Arvo Viltrop^3^, Dirk Höper^1^, Carola Sauter-Louis^1^, Martin Beer^1^ and Sandra Blome^1*^

^1^ Friedrich-Loeffler-Institut, Suedufer 10, 17493 Greifswald – Insel Riems, Germany

^2^ Estonian Veterinary and Food Laboratory, Kreutzwaldi 30, 51006 Tartu, Estonia

^3^ Institute of Veterinary Medicine and Animal Sciences, Estonian University of Life Sciences, Kreutzwaldi 62, 51014 Tartu, Estonia

**Corresponding author:** Dr. Sandra Blome, sandra.blome@fli.de

**supplementary Fig. 1a: Trial B; qPCR results of fecal swabs** cq values graphed as line and scatter plot

**supplementary Fig. 1b: Trial B; qPCR results of oral swabs** cq values graphed as line and scatter plot

**
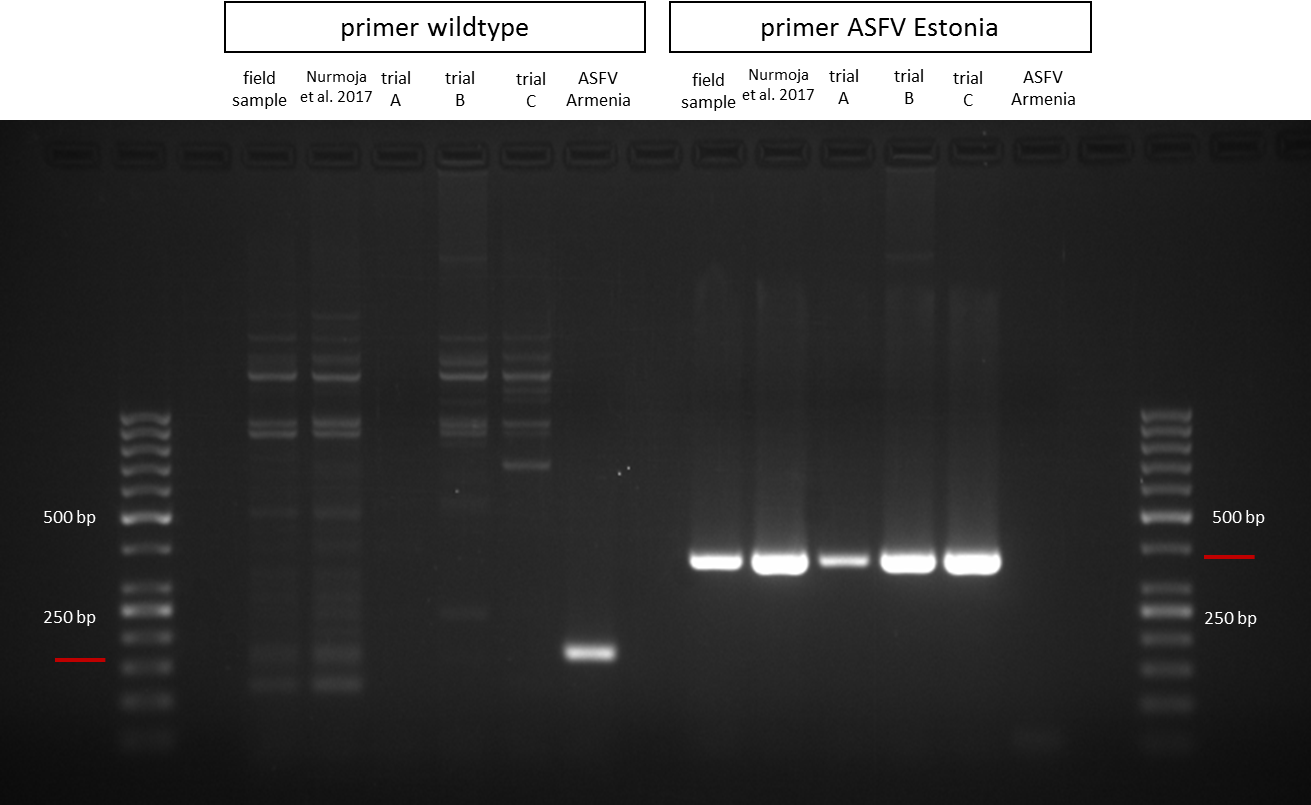
**

**supplementary Fig. 2: PCR products of the screening for the deletion site.** The amplicon length of the wildtype primers was 172bp and the amplicon length of the deletion site primers was 375bp. Representative trial samples of the trials were chosen and ran on one single gel exposed as a whole.

**supplementary Tab. 1: qPCR results of tissue samples of trial A-C** cq values detected in different tissue samples; higher and lower genome loads highlighted

**supplementary Tab. 2: virus isolation results of tissue samples** pos + indicates weakly positive, pos ++ strongly positive virus isolation results; neg marks negative virus isolation results
